# Natural products from microbes associated with insects

**DOI:** 10.3762/bjoc.12.34

**Published:** 2016-02-19

**Authors:** Christine Beemelmanns, Huijuan Guo, Maja Rischer, Michael Poulsen

**Affiliations:** 1 Leibniz Institute for Natural Product Research and Infection Biology e.V., Beutenbergstrasse 11a, 07745 Jena, Germany; 2 Centre for Social Evolution, Section for Ecology and Evolution, Department of Biology, University of Copenhagen, Universitetsparken 15, Building 3, 1st floor, 2100 Copenhagen East, Denmark

**Keywords:** biosynthesis, chemical ecology, natural products, secondary metabolism, structure elucidation, symbiosis

## Abstract

Here we review discoveries of secondary metabolites from microbes associated with insects. We mainly focus on natural products, where the ecological role has been at least partially elucidated, and/or the pharmaceutical properties evaluated, and on compounds with unique structural features. We demonstrate that the exploration of specific microbial–host interactions, in combination with multidisciplinary dereplication processes, has emerged as a successful strategy to identify novel chemical entities and to shed light on the ecology and evolution of defensive associations.

## Introduction

Although natural products represent the most consistently successful drug leads [1–2], many pharmaceutical companies eliminated their natural product research during the past decades due to diminishing returns from this discovery platform. Instead they intensely focused on screening efforts and combinatorial chemistry to find and develop novel drug candidates.

This approach of target-focused screening of synthetic compound libraries to counteract a declining number of new antibiotic entities in the drug development pipeline has largely failed [3], and the current poor repertoire represents a ”ticking time bomb”. Societies face, as a consequence of the rapid globalization and intensive use of antibiotics, an increasing threat of multidrug-resistant pathogens, which are responsible for the growing numbers of lethal infections [4–5]. The urge to discover novel lead-like antibiotic compounds and to refill the industrial antibiotic pipeline to meet current and future societal challenges has never been greater [6].

**Figure 1:**
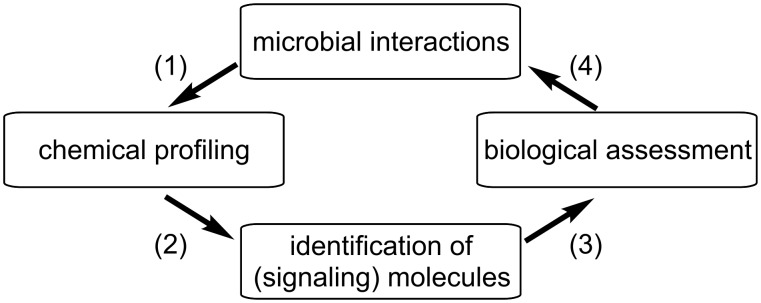
Flow chart of the typical characterization of chemical signals from microbial interactions. (1) Chemical profiling of microbial interactions using analytical techniques. (2) Dereplication leads to potentially new small molecules. (3) Optimization of the isolation protocol based on biological assessment of the activity of the isolated compounds. (4) General conclusions about ecological role and evolution of interactions.

Nowadays the major drawback of natural products research and drug discovery represents the re-isolation of known compounds and the random nature – in terms of organisms explored – by which this research is performed. Most compounds are still isolated from random sources and tested against random targets to find more or less useful bioactivities. More rational approaches are necessary to enhance the efficacy, efficiency, and speed of drug discovery in general and antibiotic discovery in particular. In recent years, the exploration of the chemical basis of specific and well-described bacteria–host or fungal–host interactions in combination with analytical dereplication processes has emerged as a powerful strategy to identify novel chemical entities (Figure 1) [7–8].

Since their initial appearance, natural products and the respective complex biosynthetic machineries have been in a constant state of evolutionary-based refinement for at least a billion years [9–11]. They function as chemical modulators and signaling molecules for intra- and interkingdom interactions such as defense, protection, behavior, virulence, and central physiological functions; thereby generating evolutionary benefits for the producer in natural habitats [12–17]. Recent developments in analytical chemistry, genome sequencing and molecular biology facilitate the analyse of minute amounts of biological material and enable a more efficient interaction-to-molecule discovery approach [18–23]. These studies also place the natural products into a genomic, regulatory, functional, and ecological context, and might allow drawing more general conclusions about the biosynthetic origins, the ecology and evolution of symbiotic associations. However, even in this ecological context natural product chemistry is highly capricious, because so far, we are not able to calculate or predict which molecular structures are responsible for a certain biological function. Despite this aspect, natural products originating from insect–microbial symbioses have a vast biochemical diversity which is a powerful resource for drug discovery [24–27].

Below we provide an overview of natural products isolated from microbial symbionts of insects, and the analytical dereplication methods when these have been applied to identify the molecules. The (potential) ecological function of the identified natural products will be discussed. We will not go into details about biosynthetic origins and assembly lines of the respective compounds, which have partially been reviewed in detail previously [28–32]. We are building on existing excellent reviews [12–17,24–26], and apologize in advance to the many researchers whose research might not be covered.

## Review

### Insects as host systems

**Figure 2:**
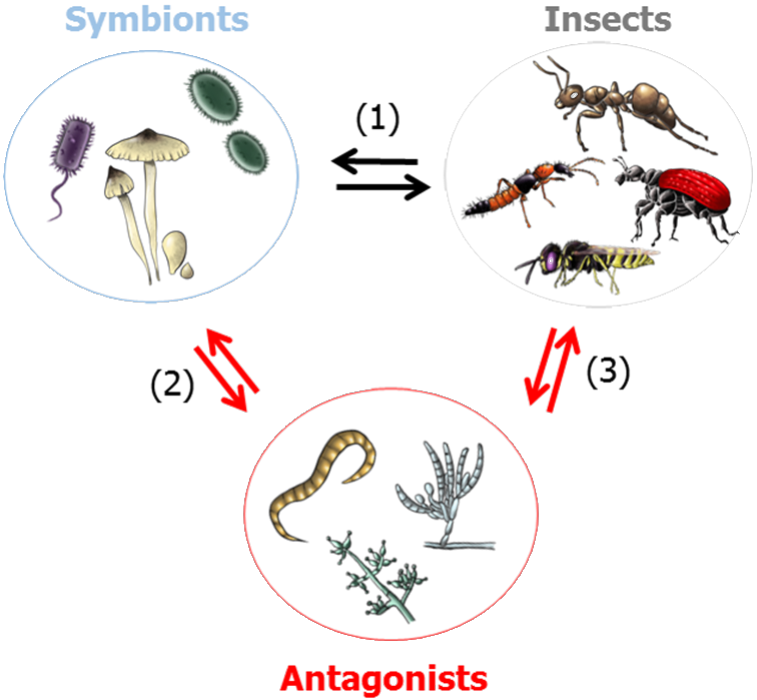
Multilateral microbe–insect interactions. (1) Insect–symbiont interactions with both partners benefiting from the interactions. (2) Antagonistic microbial interactions (e.g., competition for nutrients and space). (3) Antagonistic microbe–insect interactions (e.g., entomopathogenic microbes).

Insects, the most diverse groups of animals on Earth [12–17], originated about 480 million years ago, at about the same time period when terrestrial plants evolved [33]. Since their initial appearance, insects have occupied almost every environmental niche while in the meantime, symbiotic and/or pathogenic microorganisms have adapted specifically to insects as host systems (Figure 2) [34–36]. As an immediate response, insects were colonized by symbiotic microorganisms that are often required by the insect host to provide necessary nutritional and immunological effectors (obligate symbiont) [37]. The microbiota may account for 1–10% of the insect biomass, implying that the insect, as well as any other higher organism, can be regarded as a multi-organismal entity [38]. Due to specialized lifestyles and feeding behavior, insects are often prone to exploitation and pathogen infestation. In particular, life in large communities (social insects), the mass provisioning of nutrients to the offspring, and the construction of brooding chambers are threatened by invading and predatory species [12–17].

As a response to these threats, many insects have evolved defensive strategies, including mechanical and behavioral defense, complex immune systems, and the use of bioactive secondary metabolites produced by residing mutualists [12–17,24–26]. The occurrence of these metabolites in often subinhibitory concentrations indicates that they might not primarily function as antimicrobials. Rather they work as signaling molecules leading to modulation of gene expression in the target organism, to alteration in factors contributing to the virulence or persistence of bacterial pathogens, or to the development and persistence of microbial communities [39–41]. Nowadays it is hypothesized that the evolution and diversification of the microbial biosynthetic machinery may have evolved secondarily in interactions with other organisms, and microbial–insect interaction and regulation mechanisms are likely to be more complex than previously expected.

### Defensive bacterial symbionts of insects

**Figure 3:**
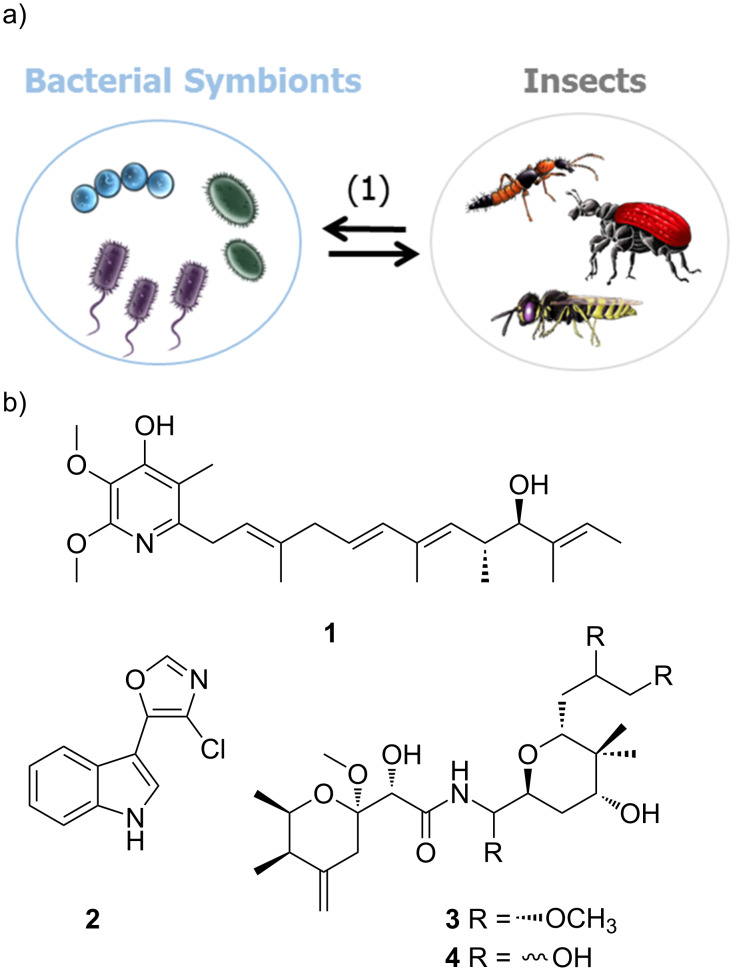
a) Interactions between bacterial (endo)symbionts and insects with both partners benefiting from the interactions (1). b) Defensive secondary metabolites isolated from bacterial symbionts: piericidin A1 (**1**), streptochlorin (**2**), pederin (**3**), and diaphorin (**4**).

Kaltenpoth and co-workers described one of the most intriguing examples of an insect–bacteria symbiosis and symbiont conferred protection [42–44]. Predatory females of the solitary digger wasp European beewolf (*Philanthus triangulum*), catch and paralyze honeybees and use the insect prey as food source for their larvae. To protect the offspring, beewolves cultivate the endosymbiont ”*Candidatus* Streptomyces philanthi” in antennal glands. By inoculation of the soil of the brood cell with the protective symbiont, beewolf females ensure that the larvae take up the symbionts from the surrounding soil while spinning the cocoon. Using high resolution mass spectrometry (HRMS) and nuclear magnetic resonance (NMR) spectroscopy, the protective secondary metabolites were identified as piericidin derivatives (e.g., piericidin A_1_ (**1**), Figure 3) and the chlorinated indole derivative streptochlorin (**2**). Imaging analysis based on a combination of laser desorption/ionization (LDI)–time of flight (TOF) mass spectrometry imaging visualized the spatial distribution of the antibiotics on the outer cocoon surface. Subsequent gas chromatography–mass spectrometry (GC–MS) analyses and expression studies revealed that the production of both antibiotics peaked within the first two weeks after cocoon spinning [45]. Although expression levels decreased shortly afterwards, the antibiotic substances were detectable on the cocoon surface for months during hibernation.

Structurally, piericidins consist of a pyridone core attached to polyene side chains of variable size, a structural and physiochemical feature of ubiquinone. Therefore, it is not surprising that piericidins are potent inhibitors of mitochondrial and bacterial NADH–ubiquinone oxidoreductase (complex I) [46]. Streptochlorin (**2**), on the other side, belongs to the natural compound class of 5-(3-indolyl)oxazoles, and has been isolated from many different (marine) Actinobacteria species. Streptochlorin and closely related derivatives have been shown to possess a variety of biological activities, such as antibiotic, antifungal and antiproliferative activity [47]. The combination of the antibiotic properties of piericidins and streptochlorin is most likely the reason for the effective inhibition of various entomopathogenic microbes, indicating a ”first chemical defense line” and ”long term prophylaxis” of *P. triangulum* ensuring protection and enhanced survival rates of the offspring.

In a similar study, a detailed chemical analysis of rove beetles (*Paederus* spp.) led to the isolation of the complex polyketide pederin (**3**), a potent toxin that can ward of natural predators such as wolf spiders [48]. The initial isolation of pederin (**3**) included the collection and chemical analysis of 250,000 beetles. Later, the true producer was found to be an endosymbiotic *Pseudomonas* sp. within the female beetle which was identified by molecular analysis of the biosynthetic gene cluster of pederin (**3**) [49–52]. Beetle larvae hatching from pederin-containing eggs were less prone to predation by wolf spiders than pederin-free larvae, indicating the ecological significance of this secondary metabolite [53]. The biosynthetic gene cluster analysis also revealed that pederin is formed by an enzyme belonging to a functionally and evolutionarily novel group termed trans-acyltransferase PKSs (trans-AT PKSs) [24,52]. The structurally related compound diaphorin (**4**) was later found in a study of the defensive symbiosis between the Asian citrus psyllid and the β-proteobacterium ”*Candidatus* Profftella armatura” [54–55]. A genome analysis of *Profftella*, which resides in a symbiotic organ called the bacteriome, revealed that 15% of the drastically reduced genome encoded horizontally acquired genes for the biosynthesis of the polyketide toxin indicating an ancient and mutually obligatory association with the host. In another model system, it was also found that the aphid symbiont, *Hamiltonella defensa*, harbors a prophage that encodes proteinaceous toxins (Shiga-like toxin, cytolethal distending toxin, YD-repeat toxin), which is believed to protect aphids from the parasitic wasp *Aphidius ervi*. [56–57].

Various other protective functions of bacterial endosymbionts have been characterized, but the molecular basis of these interactions still remains elusive. Examples include defensive bacterial symbionts of aphids and their activity against entomopathogenic fungi [58], and the defensive character of *Spiroplasma* species (Tenericutes phylum) associated with *Drosophila* species [59–60].

### Defensive bacterial symbionts of fungus-growing insects

**Figure 4:**
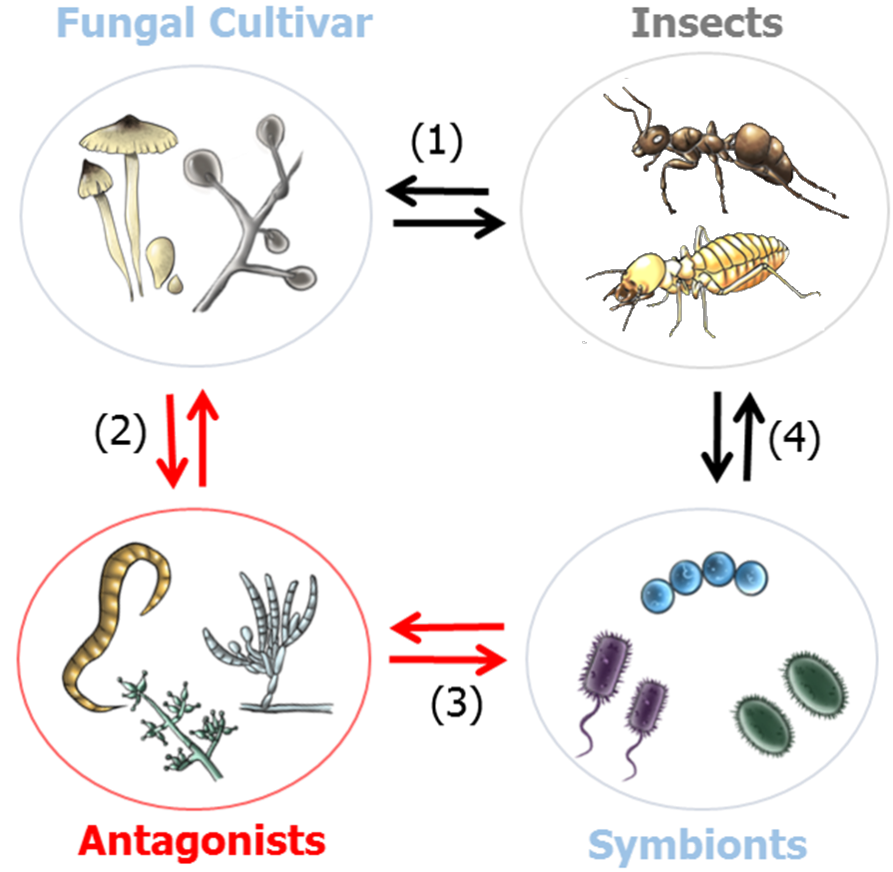
Multilateral microbial interactions in fungus-growing insects. (1) Insect cultivar: protects and shares habitat and nutrients. (2) Cultivar antagonist: competition for nutrients and habitat. (3) Antagonist mutualist: competition for nutrients and habitat; detrimental infestation by antagonist. (4) Symbiont insect: (beneficial) coexistence by sharing and protecting habitat and nutrients.

Insects, such as ants [61–62], termites [63], beetles [64], and even some bees [65] engage in fungi culture [66]. Fungus-growing insects create fungal gardens underground or in wooden galleys in which they grow an obligate food fungus that they supply with organic matter (Figure 4). The nutrient-rich fungus gardens are prone to exploitation by parasitic microorganisms, nematodes and other predators (e.g., other insects), rendering a high selective pressure on the insect to evolve effective (chemical) defenses [12–13,67–68].

#### Fungus-growing ants

**Figure 5:**
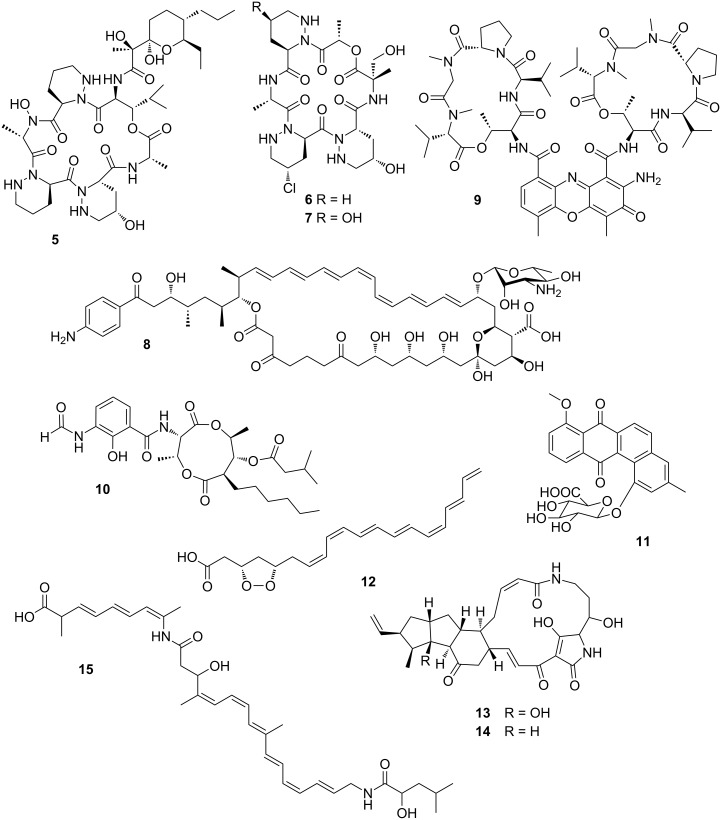
Small molecules (chemical mediators) play key roles in maintaining garden homeostasis in fungus-growing insects: dentigerumycin (**5**), gerumycin A (**6**), gerumycin C (**7**), candicidin D (**8**), actinomycin D (**9**), antimycin A1 (**10**), pseudonocardone B (**11**), mycangimycin (**12**), frontalamide A (**13**), frontalamide B (**14**), and bacillaene A (**15**).

One of the best-studied defensive symbiosis are leaf-cutting ants [69–70]. The symbiotic relationship between ants and fungus is particularly challenged by invading fungal species such as *Escovopsis*, *Fusarium*, and *Trichoderma* (Ascomycota). To clean the garden, ants apply mechanical grooming [71] and secrete antimicrobial compounds, such as 3-hydroxydecanoic acid, from their metapleural glands [72]. As a second line of defense, the ants are associated with protective Actinobacteria belonging in most attine ant genera to the genus *Pseudonocardia*, which grow on species-specific areas of the cuticle [73–76]. In vitro bioassay-guided screening of one of the *Pseudonocardia* symbionts afforded the antimicrobial cyclic depsipeptide dentigerumycin (**5**) that selectively inhibits the growth of the nest parasite *Escovopsis* but not the ants’ mutualistic fungus at micromolar concentrations [77]. Dentigerumycin bears an unusual amino acid core skeleton including three piperazic acids, β-hydroxyleucine, *N*-hydroxyalanine, and a polyketide-derived moiety with a pyran ring. A follow-up study via genomic analysis and metabolomic profiles revealed that piperazic acid-containing cyclic depsipeptides are very common in this ecological niche of ant-associated bacteria. Fermentation and purification of metabolite extracts of three ant-associated *Pseudonocardia* derived from different geological places (Panama and Costa Rica) lead to the isolation of additional dentigerumycin-like molecules (e.g., gerumycin A (**6**) and gerumycin C (**7**), Figure 5) [78].

Gerumycins lack the polyketide-derived moiety, but contain e.g. a modified piperazic acid moiety carrying an additional chlorine and/or hydroxy substituent. In contrast to dentigerumycin, gerumycins do not exhibit significant antifungal activity in vitro against dentigerumycin-sensitive *Escovopsis* strains. A detailed biosynthetic analysis of gerumycins revealed that the biosynthetic gene clusters are encoded within variable genetic architectures and greatly differ between the three producing bacteria that it is not possible to deduce an evolutionary relation [78]. Over the last decade, the chemical investigation of *Pseudonocardia* and other Actinobacteria from fungus-growing ant species has led to the isolation and identification of many, including known, antimicrobial compounds. Among the reported structures are candicidin derivatives (e.g., candicidin D (**8**)) [79–81], actinomycin derivatives (e.g., actinomycin D (**9**)) [82], antimycin derivatives (e.g., antimycin A1 (**10**)), and novel quinones (e.g., pseudo-nocardone B (**11**)) [83] as depicted in Figure 5. This reflects the defensive role of Actinobacteria against fungus garden invaders and demonstrates their enormous biosynthetic potential as producers of antimicrobial compounds. Despite intensive research efforts, the specificity and evolutionary history of the ant–*Pseudonocardia* association still remains controversial [84–85]. It has been hypothesized that many of the isolated soil-dwelling Actinobacteria may have also been recruited from the environment by horizontal transmission, without having tight evolutionary bonds to the insect host.

#### Fungus-growing beetles

Bark beetles like the Southern Pine beetles (*Dendroctonus frontalis*) are responsible for widespread destruction of trees in parts of the United States [64]. They engage in an obligate symbiosis with the fungus *Entomocorticium* sp. A (Ascomycota), which serves as nutrition for the beetle larvae, but also eventually causes the death of the tree. To propagate the fungus, adult beetles carry *Entomocorticium* sp. A in a specialized storage compartment called a mycangium from which the galleries within the inner bark of the host pine tree, housing the beetle larvae, are inoculated. The symbiosis is threatened by an antagonistic fungus *Ophiostoma minus*, which is able to overgrow *Entomocorticium* sp. A. To counteract this threat, *D. frontalis* house defensive bacterial symbionts within the galleries as well as inside the mycangia that appear to suppress the antagonistic fungus *Ophiostoma*.

Using symbiont pairing bioassays and chemical analysis one of the major isolates *Streptomyces thermosacchari* was shown to produce the fungicide mycangimycin (**12**), which inhibits the growth of the antagonist *O. minus*. Mycangimycin is an unusual carboxylic acid derivative with an endoperoxide unit and a conjugated heptaene moiety [86–87]. Subsequent chemical analysis of another *Streptomyces* strain associated with the southern pine beetle led to the discovery of two new members of polyketide-derived polycyclic tetramate macrolactams named frontalamides A (**13**) and B (**14**) (Figure 5) [88–89], which also displayed negative effects on the growth of the antagonistic fungus *O. minus*. By genetic analysis and manipulation of the producing *Streptomyces* strain the respective biosynthetic gene cluster could be identified. It encodes a hybrid polyketide synthase–non-ribosomal peptide synthase (PKS–NRPS), and resembles iterative enzymes normally only found in fungi. Subsequently, genomes of phylogenetically diverse bacteria from various environments were screened for the biosynthetic pathways of frontalamide-like compounds using a degenerate primer-based PCR screen. The respective gene clusters were broadly distributed in environmental Actinobacteria and the presence of the compounds was confirmed by chemical analysis of the bacterial cultures by LC–MS. Once again, these examples show that antibiotic-producing Actinobacteria may be commonly maintained as defensive microbes.

#### Fungus-growing termites

**Figure 6:**
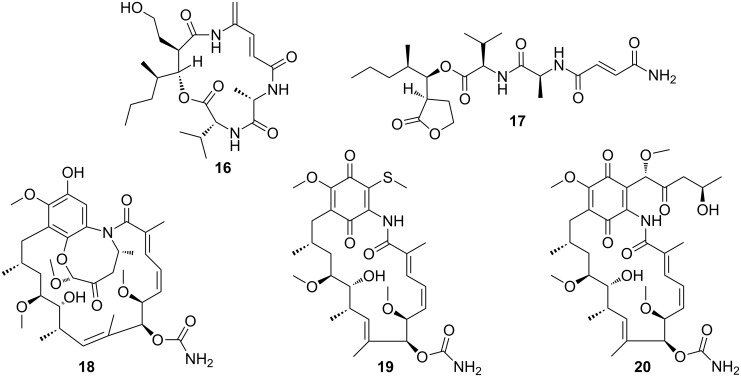
Secondary metabolites isolated from Actinobacteria from fungus-growing termites. Microtermolide A (**16**), microtermolide B (**17**), natalamycin A (**18**), 19-*S*-methylgeldanamycin (**19**), and 19-[(1*S*,4*R*)-4-hydroxy-1-methoxy-2-oxopentyl]geldanamycin (**20**).

The monophyletic termite subfamily Macrotermitinae propagates a basidiomycete fungal cultivar *Termitomyces*, which serves as a major food source for the termite colony [90]. The domestication of *Termitomyces* facilitates an increase in carbohydrate decomposition capacity relative to that of other higher termites [91]. In turn, the termites cultivate and clean the fungus gardens; thus, protecting them from infestation by invasive species (e.g., mycoparasitic *Trichoderma* species). Despite targeted efforts, strong evidence for defensive microbial symbionts has remained elusive [92]. Only one study showed that the fungus-growing termite *Macrotermes natalensis* harbors a *Bacillus* strain, which produces a single major antibiotic, bacillaene A (**15**) (Figure 5), that inhibits putatively competitive or antagonistic fungi of *Termitomyces* suggesting a defensive property [93]. In various other studies, *Streptomyces* have been isolated from fungus-growing termite workers and combs, and some of these have been investigated for their chemical potential despite their so far largely undefined role in the symbiosis. Bugni and co-workers prioritized *Streptomyces* isolates from fungus-growing termites based on a HRMS-based principle component analysis (PCA) to rapidly identify unique natural product producers [94]. Based on this strategy, Clardy and co-workers then performed detailed chemical investigations of strains with an unique metabolomic profile, which led to the isolation, characterization, and reassignment of microtermolides A (**16**) and B (**17**) (Figure 6), products by an unusual hybrid non-ribosomal–polyketide pathway [95]. In a follow-up study, a *Streptomyces* isolate with exceptional high antifungal activity was investigated, and an unusual geldanamycin-derived natalamycin A (**18**), 19-*S*-methylgeldanamycin (**19**), and a geldanamycin analog with an unusual side chain modification (**20**) were isolated (Figure 6) [96]. The structure of **18** was elucidated using a combination of NMR spectroscopy, X-ray crystallography and additional quantum chemical NMR calculations.

### Bacterial mutualists

*Streptomyces* and other Actinobacteria are well adapted to living in symbiosis with invertebrates, and have been isolated from many different parts of different insect species [12]. To further illuminate the importance of Actinobacteria as producers of valuable small molecules, we provide below additional examples of novel bioactive secondary metabolites originating from Actinobacteria–insect interactions, despite lack of clarity regarding the specificity and evolutionary history of these associations [97–99].

**Figure 7:**
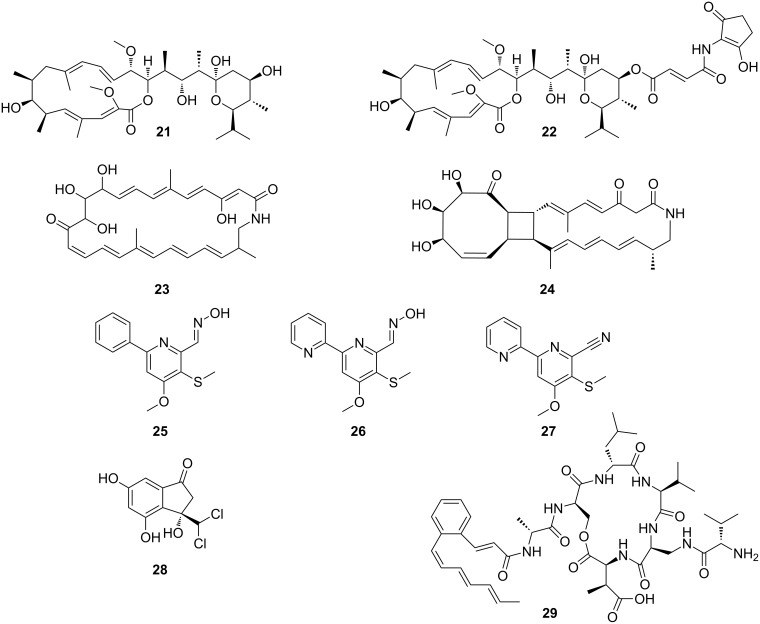
Secondary metabolites from bacterial mutualists of solitary insects. Bafilomycin A1 (**21**), bafilomycin B1 (**22**), sceliphrolactam (**23**), tripartilactam (**24**), coprismycin A (**25**), collismycin A (**26**), dipyridine SF2738D (**27**), tripartin (**28**), and coprisamide A (**29**).

As described by Poulsen et al. a large number of morphologically, phylogenetically, and chemically diverse *Streptomyces* strains were isolated from two solitary wasp species (*Sceliphron caementarium* and *Chalybion californicum*, Hymenoptera, Sphecidae) [100]. Based on a pre-screening of bacterial extracts, the detailed chemical analysis of selected strains revealed not only a broad range of known bioactive compounds, such as bafilomycins (e.g., bafilomycin A1 (**21**) and B1 (**22**), Figure 7), but also a novel polyunsaturated and polyoxygenated 26-membered macrolactam named sceliphrolactam (**23**) (Figure 7) [101]. Sceliphrolactam showed strong antifungal activity against amphotericin B-resistant *Candida albicans*, but its functional role in vivo remains enigmatic.

In another study, Oh and co-workers chemically investigated a diverse population of Actinobacteria from the indigenous soil-dwelling Korean dung beetle (*Copris tripartitus*), its larvae and dung balls [102–103]. Dung beetles are prime contributors to the cyclic breakdown of organic waste material, and their life cycle is tightly dependent on herbivore faeces [104–105]. Based on unique metabolomic profiles (UV chromatogram) and HRMS data, several of the isolated *Streptomyces* strains were selected for large scale fermentation. Detailed chemical analysis of an organic culture extract led to the isolation of a new tricyclic macrolactam named tripartilactam (**24**) [103]. Tripartilactam (**24**) contains an unprecedented cyclobutane moiety, which links the 8- and 18-membered rings, and it is most likely derived from a photochemically [2 + 2] cycloaddition reaction of the corresponding macrocyclic 26-membered lactam precursor. Although compound **24** lacks any significant antimicrobial and anticancer activity, it was shown to act as a Na^+^/K^+^ ATPase inhibitor.

Subsequent studies by the same group lead to the isolation of phenylpyridines (e.g., coprismycin A (**25**)), dipyridines (e.g., collismycin A (**26**), SF2738D (**27**)) [102], and a dichlorinated indanone tripartin (**28**) [106]. Recently, the same group isolated new cyclic heptapeptides, named coprisamides (e.g., coprisamide A (**29**)) from a *Streptomyces* strain isolated from the gut of *C. tripartitus*. The cyclic heptapeptides contain unusual amino acid units (e.g., β-methylaspartic acid and 2,3-diaminopropanoic acid) and a previously unreported 2-heptatrienyl cinnamoyl chain unit [107]. Dung beetle larvae are prone to bacterial and fungal infestations during their development inside the faeces balls. Although the direct involvement of defensive microbial symbionts has not been described yet, the presence of highly productive Actinobacteria might provide an indirect protection against parasites and pathogens as suggested in the termite symbiosis.

### Fungal symbionts

Fungi co-evolved with various different insects over millions of years, thereby serving as a food source to fungal grazers, or competing with saprophagous insects, and attacking insects as hosts for growth and reproduction [108]. The cross-kingdom interactions and long-time co-evolution are assumed to be responsible for the genetic accumulation of biosynthetic gene clusters encoding for bioactive secondary metabolites. The respective natural products are predicted to play key roles as chemical signals or virulence factors mediating the interactions with the respective insect host [108–111].

**Figure 8:**
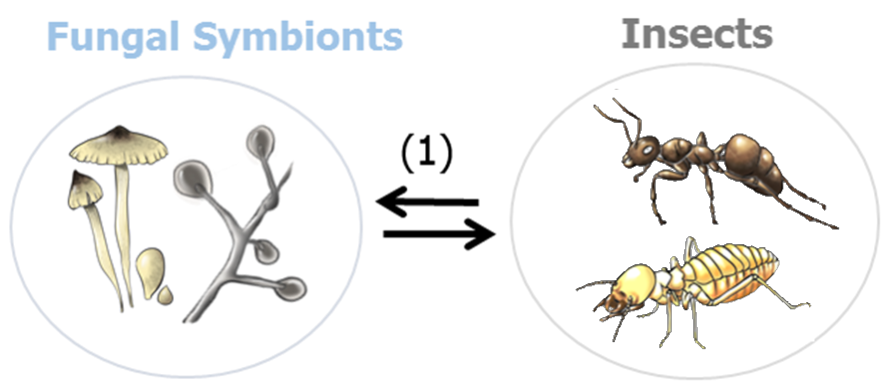
Beneficial interactions (1) between fungal symbionts and insects.

Despite the fact that a few examples exist, fungi as (defensive) symbionts have not nearly been explored to the same extent as bacterial protagonists, which is surprising as fungi have a vast biosynthetic potential and are a rich source of antibiotics (Figure 8).

**Figure 9:**
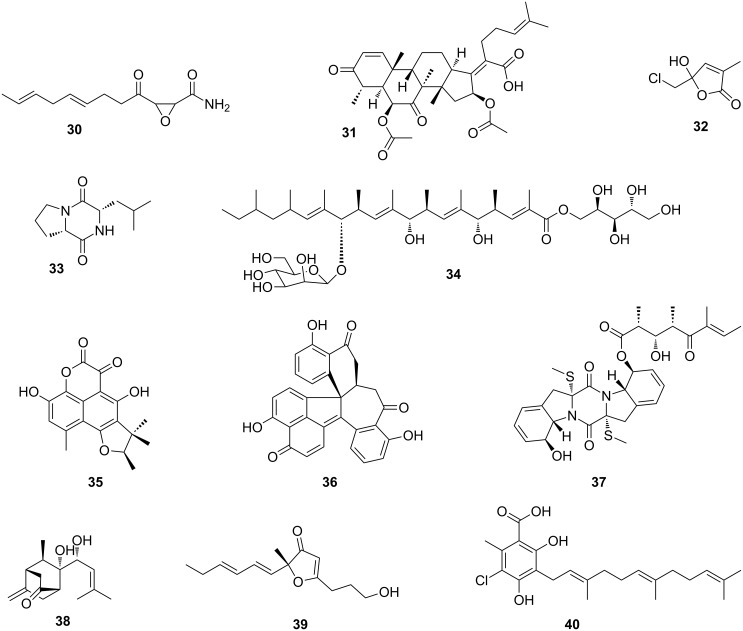
Secondary metabolites isolated from fungal symbionts. Cerulenin (**30**), helvolic acid (**31**), lepiochlorin (**32**), cyclo-(L-Pro-L-Leu) (**33**), bionectriol A (**34**), (+)-scleroderolide (**35**), dalesconol A (**36**), boydine B (**37**), boydene A (**38**), paraconfuranone A (**39**), and ilicicolinic acid A (**40**).

As early as 1982, Nakashima et al. investigated the fungal cultivar (*Fusarium* sp.) of the ambrosia beetle *Euwalecea validus*. The chemical analysis of culture extracts revealed the antifungal secondary metabolites cerulenin (**30**) and the nortriterpenoid helvolic acid (**31**) (Figure 9), which inhibit the growth of mold fungi in vitro and are assumed to suppress bacterial contaminations [112]. Slightly earlier, in 1979, Nair et al. had described the isolation of an antibacterial chlorinated lactol, lepiochlorin (**32**), from liquid cultures of a *Lepiota* species, a fungus cultivated by fungus-growing ants (*Cyphomyrmex costatus*) [113]. Nearly twenty years later, Clardy and co-workers explored the symbiotic interactions between the fungus *Tyridiomyces formicarum* of the fungus-growing ant *Cyphomyrmex minutus*, as part of the seminal “biorationale" approach in the search for novel compounds. The fungus is unique among the attine fungi because it grows as a yeast form (unicellular) and not in the mycelial form which is typical for all other attine ant fungi. The fungus was found to produce several antifungal diketopiperazines (e.g., **33**) [114]. In another study, also reported by Clardy and co-workers, the secondary metabolite profile of the symbiotic fungus *Bionectria* sp. associated with the fungus-growing ant *Apterostigma dentigerum*, was investigated [115]. Again, a chemical analysis of an organic culture extract led to the isolation of a new polyketide bionectriol A (**34**), a glycosylated, polyunsaturated polyol, with so far undetermined ecological function. More recently, Wang et al. showed that the solitary leaf-rolling weevil *Euops chinensis* (Attelabidae) undergoes a protofarming symbiosis with the polysaccharide-degrading *Penicillium herquei* (family *Trichocomaceae*), which is planted on leave roles containing eggs and larvae to protect the offspring. *P. herquei* was shown to produce the antibiotic polyketide (+)-scleroderolide (**35**), which can inhibit the growth of several bacterial and fungal pathogens in competition assays on plates and keeps larval brood chambers free of other microbes [116–117].

Although the ecological roles of the compounds produced by the investigated fungi remain elusive, the following examples show that associated fungi are valuable sources for novel bioactive secondary metabolites with high pharmacological potential.

In 2008, Tan and co-workers discovered the unusual polyketide dalesconol A (**36**) from extracts of the fungus *Daldinia eschscholzii* isolated from the gut of the mantis *Tenodera aridifolia* [118–119]. Additional insights into the dalesconol biosynthesis was gained from a characterization of minor dalesconols and biosynthetic intermediates only present in chemical extracts prepared from a large-scale fermentation. The ascomycete fungus *Pseudallescheria boydii*, isolated from the gut of the larvae of the beetle *Holotrichia parallela*, showed also a broad range of bioactive secondary metabolites including epipolythiodioxopiperazines, named boydines (e.g., boydine B, (**37**)) [120]. Boydines significantly inhibit clinically relevant anaerobic bacterial strains (e.g., *Bifidobacterium* sp., *Veillonella parvula*, *Anaerosterptococcus* sp., *Bacteroides vulgatus*, and *Peptostreptococcus* sp.), suggesting a potential ecological role as defensive symbiont in addition to interesting pharmacological properties. Further analysis of the same fermentation extracts afforded boydenes (e.g., boydene A, (**38**)), sesquiterpenes with an unprecedented carbon skeleton that are most likely built up by an enzymatic Aldol addition.

In a similar example, new cytotoxic furanone analogues (e.g., paraconfuranone A (**39**)) were obtained from the fungus *Paraconiothyrium brasiliense* isolated from the gut of the grasshopper *Acrida cinerea* [121]. Antibacterial ilicicolinic acids (e.g., ilicicolinic acid A (**40**)) were detected in a fungus *Neonectria discophora* isolated from a soil-feeding and wood-damaging termite nest (*Nasutitermes corniger*) in the North Amazon (French Guiana). Ilicicolinic acids show good inhibitory effects against several human pathogens [122].

### Entomopathogenic fungi

**Figure 10:**
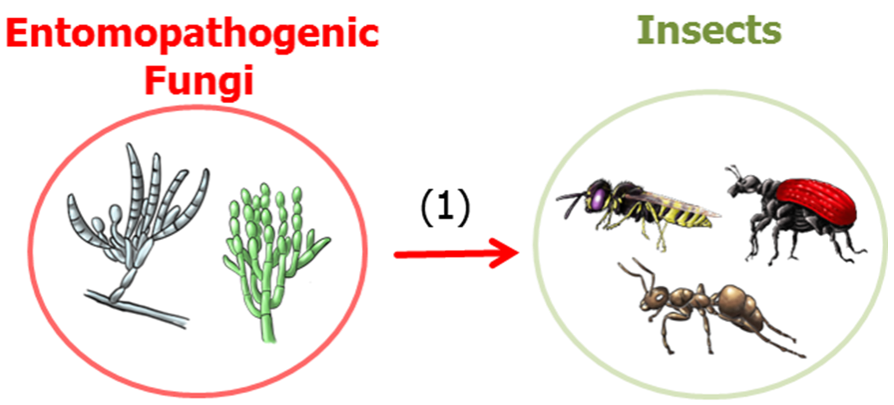
Predatory interactions, (1) entomopathogenic fungi use insect as prey.

More than 700 known fungal species from 100 genera have adopted an entomopathogenic lifestyle (Figure 10) [123–124]. Entomopathogenic fungi release infective spores which attach to the insect cuticle; once the spore germinates, the developing hyphae penetrate the insect integument and start the infection process. Apart from a variety of secreted proteases that digest the chitin-containing cuticle of the insect, secreted toxic metabolites are assumed to assist in overcoming host defenses and killing the host. Some entomopathogenic species, such as *Beauveria bassiana* and *Metarhizium anisopliae*, have a broad host range encompassing over 1,000 insect species from more than 50 different insect families. These fungi are used as biocontrol agents for invertebrate pest control, a commercial alternative to chemical pesticides [125–127]. Other entomopathogenic fungi, such as different *Cordiceps* species, are also known to be prolific producers of highly active secondary metabolites, but with a relatively narrow host range and geographic distribution [108,124]. Recent comparative genomic analyses of *Metarhizium* sp. and *Beauveria* sp. indicate that over 80% of the genes associated with putative secondary metabolites have no identified specific products, and even sequences are unique to this group of organisms [124]. Despite the enormous chemical potential, only a few studies to date have unequivocally demonstrated the exact role of the respective compounds. Here, we briefly summarize compounds for which an ecological role has been identified.

**Figure 11:**
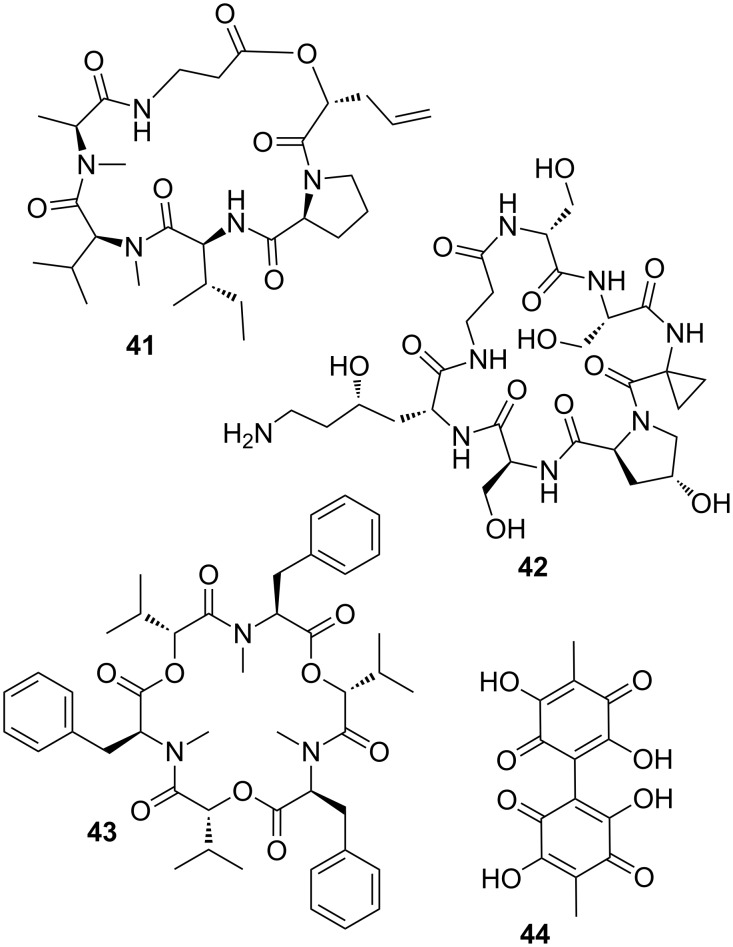
Entomopathogenic fungi use secondary metabolites as insecticidal compounds to kill their prey. Destruxin A (**41**), serinocyclin A (**42**), beauvericin (**43**), and oosporein (**44**).

One of the most prominent secondary metabolites of *M. anisopliae* are the cyclic hexadepsipeptides named destruxins (e.g., destruxin A (**41**), Figure 11). Destruxins are composed of an α-hydroxy acid and five amino acid residues, and they exhibit a wide range of interesting biological properties, such as insecticidal, cytotoxic, and moderate antibiotic activity [128]. The secretion of destruxins is weakly correlated to fungal virulence and insecticidal activity, because injection, ingestion or topical application of these compounds resulted in tetanic paralysis in many insects, caused by destruxin-mediated opening of calcium channels and resulting membrane depolarization.

In another study, the cyclic heptapeptide serinocyclins (e.g., serinocyclin A (**42**)) were isolated from conidia harvested on agar surface cultures of *M. anisopliae*, a commercial biocontrol product called Green Muscle [129]. Serinocyclin A contains several non-proteinogenic amino acids. Among them are the uncommon 1-aminocyclopropane-1-carboxylic acid, (2*R*,4*S*)-4-hydroxylysine, and the more frequently encountered hydroxyproline, β-alanine, and D-serine. Due to the presence in conidia, serinocyclines have also been hypothesized to play a role in the virulence of *M. anisopliae*.

Chemical analysis of the entomopathogenic fungus *B. bassiana* yielded beauvericin (**43**), a depsipeptide with alternating methylphenylalanyl and hydroxyisovaleryl residues. Beauvericin has antibacterial, antifungal, and insecticidal activities, in addition to its potent cytotoxic activity against human cell lines [130]; attributes which indicate a crucial role in the infection process. The red 1,4-bibenzoquinone derivative oosporein (**44**) was first identified in the 1960s [131], and exhibits similar antibiotic [132], antiviral [133], antifungal [134], and insecticidal activities [135]. Oosporein (**44**) production in *B. bassiana* is correlated to the fungal virulence due to the inhibition of host immunity, which facilitates fungal propagation in insects [136].

In summary, entomopathogenic fungi are rich in secondary metabolite gene clusters, some of which have been genetically characterized. However, the vast majority of the encoded compounds, as well as their biological role(s) remain uncovered [137]. In light of the rapidly declining costs for -omic technologies, in vivo infection studies coupled with methods such as RNA sequencing, can lead to further insights into the role and expression levels of potentially new secondary metabolites.

## Conclusion

Insects provide experimentally tractable and cost-effective model systems to investigate the evolutionary development and chemical basis of animal–bacterial interactions, and symbiosis in particular. Bacterial and fungal symbionts represent an extraordinary discovery opportunity for both biology and chemistry. Studying these interactions will shed light on equivalent processes in other animals, including humans. The in-depth investigations of a small number of insect–microbe interactions have already led to the discovery of a number of secondary metabolites with new and structurally diverse chemical core structures. Unfortunately, the identification of chemical mediators has so far been mainly restricted to in vitro analyses, but efforts should be directed towards identifying the presence and activity of candidate compounds in situ. The examination of bacterial secondary metabolisms and the respective small molecules secretome, can give insights into the up or down-regulation of (cryptic) biosynthetic pathways. This in turn can lead to the discovery of new metabolic pathways that would otherwise be silent or undetected under typical laboratory cultivation conditions. In recent years many successful analytical methods including UHPLC–DAD and UHPLC–MS-based techniques, imaging mass spectrometry (IMS) [138–139] and high resolution NMR systems have been developed and optimized [7,18]. These technologies allow the identification in minute concentrations of the chemical entities moderating insect–microbial interactions and at least partially eliminate the need for bioassay-guided fractionation for the identification of key compounds. We are still scratching the surface of the chemical potential of the microbial world, but chemical investigations of microbial interactions will undoubtedly expand the list of new bioactive secondary metabolites in the near future.
